# Validation of positional candidates *Rps6ka6* and *Pou3f4* for a locus associated with skeletal muscle mass variability

**DOI:** 10.1093/g3journal/jkae046

**Published:** 2024-04-05

**Authors:** Konstantinos Lekkos, Afra A Bhuiyan, Abdullah M K Albloshi, Paige M Brooks, Thomas M Coate, Arimantas Lionikas

**Affiliations:** School of Medicine, Medical Sciences and Nutrition, University of Aberdeen, Aberdeen AB25 2ZD, UK; School of Medicine, Medical Sciences and Nutrition, University of Aberdeen, Aberdeen AB25 2ZD, UK; School of Medicine, Medical Sciences and Nutrition, University of Aberdeen, Aberdeen AB25 2ZD, UK; Department of Anatomy and Histology, School of Medicine, University of Albaha, Alaqiq 65779, Saudi Arabia; Department of Biology, Georgetown University, Washington, DC 20057, USA; Department of Biology, Georgetown University, Washington, DC 20057, USA; School of Medicine, Medical Sciences and Nutrition, University of Aberdeen, Aberdeen AB25 2ZD, UK

**Keywords:** RSK4, myogenesis, muscle fibers

## Abstract

Genetic variability significantly contributes to individual differences in skeletal muscle mass; however, the specific genes involved in that process remain elusive. In this study, we examined the role of positional candidates, *Rps6ka6* and *Pou3f4*, of a chromosome X locus, implicated in muscle mass variability in CFW laboratory mice. Histology of hindlimb muscles was studied in CFW male mice carrying the muscle “increasing” allele C (*n* = 15) or “decreasing” allele T (*n* = 15) at the peak marker of the locus, rs31308852, and in the *Pou3f4^y/−^* and their wild-type male littermates. To study the role of the *Rps6ka6* gene, we deleted exon 7 (*Rps6ka6-ΔE7*) using clustered regularly interspaced palindromic repeats-Cas9 based method in H2Kb myogenic cells creating a severely truncated RSK4 protein. We then tested whether that mutation affected myoblast proliferation, migration, and/or differentiation. The extensor digitorum longus muscle was 7% larger (*P* < 0.0001) due to 10% more muscle fibers (*P* = 0.0176) in the carriers of the “increasing” compared with the “decreasing” CFW allele. The number of fibers was reduced by 15% (*P* = 0.0268) in the slow-twitch soleus but not in the fast-twitch extensor digitorum longus (*P* = 0.2947) of *Pou3f4^y/−^* mice. The proliferation and migration did not differ between the *Rps6ka6-ΔE7* and wild-type H2Kb myoblasts. However, indices of differentiation (myosin expression, *P* < 0.0001; size of myosin-expressing cells, *P* < 0.0001; and fusion index, *P* = 0.0013) were significantly reduced in *Rps6ka6-ΔE7* cells. This study suggests that the effect of the X chromosome locus on muscle fiber numbers in the fast-twitch extensor digitorum longus is mediated by the *Rps6ka6* gene, whereas the *Pou3f4* gene affects fiber number in slow-twitch soleus.

## Introduction

Skeletal muscle tissue is responsible for vital functions such as locomotion and respiration. Additionally, it plays a role in maintenance of glucose homeostasis and thermoregulation, serves as a reservoir of amino acids during critical illness, and protects the bones and viscera. Reduced muscle mass due to aging and/or disease could affect these functions and lead to frailty, risk of falls and factures, and loss of independence (Cruz-Jentoft *et al*. 2010). There are substantial individual differences in muscle mass even before the onset of aging-related muscle mass decline. Heritability estimates indicate that a significant proportion of individual muscle mass differences, around 30–60%, are attributed to genetic variability (Silventoinen *et al*. 2008; Hernandez Cordero *et al*. 2019). While reverse genetics studies identified several key genes involved in the regulation of myogenesis (Rudnicki *et al*. 1992, 1993; Bladt *et al*. 1995; Seale *et al*. 2000; Millay *et al*. 2013; Bi *et al*. 2017) and muscle growth (McPherron *et al*. 1997), it remains unclear if naturally occurring variants of the same key genes could account for the variation in muscle mass and function observed in the population. A recent genome-wide association study (GWAS) indicated that most key genes implicated in regulation of myogenesis and muscle growth reside outside of ∼180 genomic loci linked to muscle mass in humans (Hernandez Cordero *et al*. 2019). Thus, a substantial pool of novel regulators of muscle mass that are responsible for the individual differences in a population remains to be determined.

The key index of muscle function, its contractile force, is positively associated with muscle mass. The muscle mass is determined by 3 main variables: the cross-sectional area of muscle fibers, fiber length, and the number of muscle fibers. The cross-sectional area of the fibers is adaptive and can change in response to the changing levels of physical activity and/or nutrition. Fiber length is a function of the size of the skeleton, increasing markedly during the axial growth of long bones and stabilizing after that (Gokhin *et al*. 2008). The number of muscle fibers appears to be the most rigid variable. In laboratory mice, the numbers of fibers are determined prenatally and remain stable for a substantial portion of the lifespan (Ontell *et al*. 1988). Similarly in humans, the number of fibers does not differ in elite body builders with years of training history compared to the age-matched controls, but body builders' fibers are over 50% larger in cross-sectional area (MacDougall *et al*. 1984). Therefore, it is important to understand whether the individual differences in muscle mass are due to the axial length differences, or an outcome of the adaptive changes in the fiber cross-sectional area, or if it results from differences in the muscle fiber number.

To investigate the role of the naturally occurring genetic variants in variability of muscle mass, we previously conducted a GWAS in outbred CFW mice (Nicod *et al*. 2016; Parker *et al*. 2016). That study identified genomic regions associated with the size of the individual muscles, albeit in limited resolution, as most of the identified regions contain more than one gene thus requiring further validation studies (Flint and Eskin 2012). The locus on chromosome X with a peak marker rs31308852 showed one of the strongest associations with the weight of fast-twitch tibialis anterior (TA), extensor digitorum longus (EDL), gastrocnemius, and plantaris muscles of CFW mice and attracted attention for the following reasons: (1) the confidence interval of the locus harbored only 3 positional candidates, *Pou3f4*, *Cylc1*, and *Rps6ka6*, and (2) all 3 genes were novel in the context of skeletal muscle development and growth. *Pou3f4* is expressed in developing, but not adult, skeletal muscle in mice (Dominov and Miller 1996) and therefore emerged as a plausible candidate gene. The *Rps6ka6* is ubiquitously expressed including in adult skeletal muscle tissue, albeit at a low level, whereas *Cylc1* is expressed only in the testes. Because of these expression patterns of the 3 candidate genes, we focused on the *Pou3f4* and *Rps6ka6* genes. The aims of the present study were: (1) to examine if variation in the chromosome X locus affects muscle weight through the number or size of muscle fibers and (2) to test which of the 2 candidate genes, *Pou3f4* and *Rps6ka6*, might be responsible for the effect of the locus on the muscle mass.

A preferred validation strategy for positional candidates is phenotyping a suitable knockout mouse model (Lionikas *et al*. 2023). However, in the instances where a knockout of the target gene does not exist, the cell cultures derived from relevant tissue offer an amenable and informative alternative (Hernandez Cordero *et al*. 2019; Chen et al. 2023). Because deletion of *Rps6ka6* gene affects early embryonic development hampering derivation of a knockout (Cox *et al*. 2010), we employed both approaches in the present study.

## Methods

### Murine model

To examine the mechanisms of the effect of the locus on chromosome X, EDL muscle samples collected from CFW mice for previously published reports (Nicod *et al*. 2016; Parker *et al*. 2016) were used in the current study. We tested the hypothesis that variation in the locus could have affected (1) the number of muscle fibers constituting the muscle, (2) the cross-sectional area of the fibers, or (3) both. Male CFW mice of ∼90 days of age were selected for immunohistochemical analyses of the fibers in EDL muscle. The selected mice carried a C allele (*n* = 15) or T allele (*n* = 15) at a peak marker, rs31308852, of the locus which were associated with increased or decreased muscle weight, respectively. The EDL muscle was used because the genotype showed the strongest association with its weight compared with the TA, gastrocnemius, or plantaris muscles (Nicod *et al*. 2016). Samples were processed as previously described (Nicod *et al*. 2016; Parker *et al*. 2016).

To test the hypothesis that the association of the locus with muscle mass could be due to the effect of *Pou3f4* variation, we studied a *Pou3f4* knockout mouse model described previously (Coate *et al*. 2012; Brooks *et al*. 2020). The *Pou3f4* knockout line (Jax, strain #036158) was maintained in accordance with the Georgetown University Institutional Animal Care and Use Committee protocol, #1147. Mice carrying the *Pou3f4* null allele were maintained on C57Bl/6N background for over 5 generations before these experiments. Since *Pou3f4* is located on the X chromosome, only males were used for these experiments to avoid possible variability in females due to X-inactivation. To generate *Pou3f4^y/−^* mice and littermate controls, *Pou3f4* heterozygous females were crossed with C57Bl/6N wild-type (WT) males (purchased from Charles River Laboratories). The Direct PCR Lysis Reagent (Viagen Biotech) was used to prepare crude DNA samples from tail biopsies, and then, the following primer sets were used for PCR genotyping: TCCTTGCTTCCTCCAGTCAGAGATC and ACGTCCAGCGGCCAACCCCTCAATG (WT) and CAATGCTGTTTCACTGGTTATG and CATTGCCCCTGTTTCACTATC (Cre). Because the target phenotype, fiber number, remains stable over a substantial portion of the lifespan (Ontell *et al*. 1988), animals of various ages were used. Mice were sacrificed between the ages of 47 and 171 days, and carcasses of *Pou3f4^y/−^* (*n* = 14) and their WT littermates (*Pou3f4^y/+^*; *n* = 15) were frozen until dissection. On the day of muscle dissection, the samples were thawed and processed as previously described (Nicod *et al*. 2016; Parker *et al*. 2016). Briefly, the EDL and soleus muscles were dissected under a microscope, snap-frozen in isopentane chilled with liquid nitrogen, and stored at −70°C until histological processing.

### Immunohistochemistry and imaging

Transverse cryosections from the middle part of the muscle belly were cut at a thickness of 10 µm with a cryotome (Leica CM1850UV) at −20°C and collected on a positively charged glass slide (SuperFrost Plus, Menzel Gläser). Sections were air dried and stored at −70°C. On the day of staining, the sections from CFW mice were blocked in 10% fetal calf serum in phosphate buffered saline (PBS) and incubated with primary antibodies against laminin 1:50 (L9393, Sigma-Aldrich) for 1–2 h followed by wash in PBS. After that, the samples were processed using the diaminobenzide (DAB) chromogenic staining kit SK-4100 (Vector Laboratories). Following incubation with biotinylated BA-100 secondary antibody (Vector Laboratories) at 1:200 concentration, samples were washed in PBS and incubated with avidin biotinylated horse radish peroxidase (HRP) reagent for 30 min. Following a wash in PBS, working solution of DAB was applied for no more than 5 min and the reaction was stopped by transferring the slides to distilled water. The secondary antibody, fluorescent Alexa goat antirabbit 594 (Invitrogen), was applied at 1:200 concentration for 1 h followed by wash in PBS. For the *Pou3f4* model, EDL and soleus muscle sections were subjected to tetrazolium reductase staining [30 min incubation in 50 mM TRIS (pH7.6), 1 mM NADH, 1 mM nitroblue tetrazolium at 37°C] and ATPase staining [following a 5 min acid preincubation, pH 4.47 (Brooke and Kaiser 1970)], respectively. After staining, Mowiol 4–88 (Sigma-Aldrich) was added over the sections and covered with a coverslip sealed with nail varnish. Slides (1 per muscle) were scanned using the Axioscan Z1 slide scanner (Zeiss) with 10× magnification objective.

### Cell culture

As no mouse *Rps6ka6* gene knockout has been reported, to test the hypothesis that *Rps6ka6* may be responsible for the effects of the X chromosome locus on muscle mass, we used the H2Kb myogenic cells. The H2Kb cell line was derived in Dr. Wells laboratory (Muses *et al*. 2011) from the EDL muscle of a *H-2K^b^*-tsA58 immortomouse (CBA/Ca *×* C57BL/10 background) originally reported by Jat and colleagues (Jat *et al*. 1991). The immortomouse genome harbors temperature-sensitive (ts) large tumor antigen (*Tag*) gene from the tsA58 strain of the simian virus 40 (SV40). The expression of the immortalizing *Tag* gene driven by mouse major histocompatibility complex H2Kb promoter can be induced *in vitro* by interferons. Importantly, H2Kb cells were derived from a male mouse donor (Muses *et al*. 2011) and therefore contain 1 X chromosome and a single copy of the *Rps6ka6* gene. A single copy of the X chromosome is advantageous for the generation of knockouts, and therefore, H2Kb cells were preferred to commonly used C2C12 myoblasts which are derived from a female mouse (Diokmetzidou *et al*. 2016) with 2 X chromosomes.

H2Kb myoblasts were maintained in growth medium containing 4 ng/mL of IFN-γ (R&D, 485-MI) in high-glucose Dulbecco's Modified Eagle's Medium (DMEM; Sigma, D6546), 20% fetal bovine serum, and 1% glutamine, at 33°C/10% CO_2_. To induce differentiation, the myoblasts were transferred to a high-glucose DMEM containing 5% horse serum and 1% glutamine and incubated at 37°C and 5% CO_2_.

### Development of *Rps6ka6-ΔE7* H2Kb clones

H2Kb cells lacking exon 7 of *Rps6ak6* (*Rps6ka6-ΔE7*) were generated using clustered regularly interspaced palindromic repeats (CRISPR)-Cas9 genome editing. Guide RNAs (gRNA) flanking *Rps6ak6* exon 7 (ENSMUSE00000152471) were designed using the Broad Institute web tool (http://portals.broadinstitute.org/gpp/public/analysis-tools/sgrna-design) and, 2 sequences (5′-GATGGGGTTAGAACATACTG-3′ and 5′-AACTGTATTAACAATGCTAA-3′) were selected. Each gRNA was ligated in the pX459 plasmid (Addgene plasmid ID 48139) using Quick Ligation Kit (NEB, M2200L). The plasmid also contains the Cas9 endonuclease sequence (Ran *et al*. 2013). Following transformation, Stellar Competent Cells (Takara, 636763) were incubated in SOC medium (Takara, 2011234A) and plated on Luria-Bertani (LB) agar plates containing 80 μg/mL carbenicillin (Fisher Scientific, BP2648-1) for the overnight incubation at 37°C. The next day, colonies were isolated and expanded in LB broth overnight at 37°C on a shaker (250 rpm). The following morning, plasmids were purified using a miniprep kit (Qiagen, 27104). H2Kb cells were then transfected following a modified protocol described by Bauer and colleagues (Bauer *et al*. 2015). Briefly, H2Kb cell were seeded at 7,500 cells per well in a 24-well plate. The next day, dual transfection (with 25 µg of each plasmid) was carried out using Lipofectamine LTX (Invitrogen, A12621) following the manufacturer's protocol. After 16 h the medium was replaced, and the cells were subjected to puromycin (Merck, 540222) selection (2 μg/mL) for 72 h. The surviving cells were expanded and subjected to clonal selection as follows. The cells were trypsinized, and cell suspension containing 1 cell per 100 μL of growth medium was prepared through serial dilutions. Single cells were seeded in a 96-well plate, and after 1 week, 35% of the wells contained single cell origin clones. After another week, 30 clones were expanded and genotyped using PCR with a pair of primers flanking the gRNA target sites (forward primer 5′- GGAGGGTGTTGGCATTCTCA-3′, reverse primer 5′-TCCACCTCTCTAGAGCTAGGC-3′). Five clones generated amplicons migrating faster than the WT allele. Sanger sequencing indicated that 3 of the clones with fast migrating amplicons lacked exon 7 (*Rps6ka6-ΔE7*). The 3 clones that showed similar growth pattern to the *Rps6ka6-ΔE7* clones at the start of the clonal selection but genotyped as WT allele (*Rps6ka6-wt*) were used as controls throughout the study.

### Proliferation assay

Proliferation of H2Kb cells was measured using the Click-iT Plus EdU AlexaFluor 549 flow cytometry assay kit (Invitrogen, C10646). Cells at 100 cells per mm^2^ were seeded in a 96-well plate and incubated overnight at 33°C/10% CO_2_. The staining started with a 3 h incubation in 10 mM EdU-containing medium. Then, the cells were washed thrice in 1% bovine serum albumin (BSA) in PBS, Click-iT fixative was added to the cells for 15 min then washed in 1% BSA in PBS, and a 15 min incubation in Click-iT saponin-based permeabilization and wash reagent followed. At the end, the Click-iT Plus reaction cocktail was applied for 30 min followed by 3 5 min washes in Click-iT saponin-based permeabilization and wash reagent. Cell nuclei were stained with 300 nM DAPI solution applied for 5 min. After 3 washes in PBS, the samples were imaged. The experiment was repeated 3 times with consecutive passages of the cells.

### Migration assay

The capacity of Rps6ka6-wt and Rps6ka6-ΔE7 myoblasts to migrate was assessed using an in vitro scratch assay as previously described (Liang *et al*. 2007). In brief, following an overnight incubation of 100,000 cells/well in a 6-well plate in growth medium, a scratch was performed on the bottom of the well longitudinally using a P200 pipette tip. The wound was left to regenerate overnight. To assess the migrative ability of myoblasts, 2 nonoverlapping images were taken immediately after the scratch was formed as well as 24 h postscratching using an inverted phase contrast light and fluorescence microscope (EVOS XL, Life Technologies). The experiment was repeated 3 times with consecutive passages of cells.

### Differentiation assay

H2Kb cells were seeded in a 12-well plate at a density of 40,000 cells/well and incubated overnight in growth medium. The following day, the cells were washed in PBS, and differentiation medium was applied. Differentiation medium was replaced 3 days later and removed after 6 days, and the cells were fixed in 4% paraformaldehyde overnight at 4°C. The fixed cells were washed in PBS and permeabilized in 0.5% Triton X-100 in PBS for 15 min followed by a PBS wash. The cells were incubated in blocking buffer (10% fetal calf serum in PBS) for 1 h followed by an incubation in antimyosin, skeletal, fast antibody (M4276, Sigma-Aldrich) in PBS (1:400) for 2 h at room temperature. After 3 washes in PBS and Tween 20 0.025%, the secondary antibody (1:400 donkey antimouse Alexa 488, ab150109, abcam) and 300 nM DAPI in PBS were applied for 2 h at room temperature. Finally, the cells were washed 3 times in PBS before imaging. The experiment was repeated 4 times following consecutive passages. Six nonoverlapping images were acquired per well (2 images top, 2 middle, and 2 bottom of the well) with an inverted phase contrast light and fluorescence microscope (EVOS XL, Life Technologies).

### Image analysis

The images were analyzed using the FIJI/ImageJ software (Schindelin *et al*. 2012). A semiautomatic process for the CFW samples involved setting the scale and splitting color channels. The laminin- and type IIA myosin-stained images were analyzed for the number and cross-sectional area of muscle fibers. The red channel image was converted to a binary image using the thresholding function and processed using the binary functions Dilate and Erode to enhance the boundaries of all muscle fibers. It was followed by the Analyze Particles function with a setting of 0.4–1.0 for circularity to exclude erroneous objects within the expected size range, 50–4,500 µm^2^, but not consistent with muscle fiber shape. The output was visually inspected. In the instances where adjacent fibers appeared incompletely separated, the connections were manually deleted before repeating the analysis. For *Pou3f4* samples, muscle fibers were counted using the Cell Counter plugin.

In the analyses of the proliferation assay images, a threshold was set to select all the EdU-positive and DAPI-positive particles in the red and blue channel images, respectively, and convert them to a binary image. Subsequently, the number of nuclei was quantified using the Analyze Particles function. The percentage of proliferating cells was quantified by dividing the number of EdU-positive particles by the number of DAPI-positive particles and multiplying by 100%.

The quantification of the migrative capacity of the myoblasts was performed as previously described (Pijuan *et al*. 2019). In brief, following duplication of the wound region, the border of the cells was identified using the “find edges” function and the image was blurred using the “gaussian blur” function on FIJI/ImageJ. Using a threshold, the area of the well not covered in cells was selected and quantified through the Analyze Particles function. The percentage open wound was calculated by dividing the area devoid of cells at 24 h postwounding by the initial wound area at 0 h postwounding and multiplied by 100%. The percentage wound closure was calculated by subtracting the percentage open wound from 100%.

In the analyses of differentiation assay images, we quantified the total number of myosin-positive cells and myosin-positive cells containing more than 2 nuclei (fusion index) using the “multi-point” tool. The counts were corrected against the total area (mm^2^) of 6 captured images per well. To measure the area of myosin-positive cells, reflecting the size of differentiating cells, a threshold was set to create a binary mask highlighting the myosin-positive cells. The Analyze Particles function was then used to calculate the average area (μm^2^) per myosin-expressing cell.

### Statistical analyses

Statistical analysis and data visualization was performed on the Prism5 software (GraphPad, version 5.04). A *t*-test was used in the analyses of histology results. A 2-way ANOVA followed by Bonferroni posttest was employed to assess the effects of genotype (2 levels) and time (3 or 4 levels) in the analyses of *in vitro* data. *P*-values are stated in-text, and the level of significance is denoted on the graphs as *P < 0.05, **P < 0.01, and ***P < 0.001. Data are presented as mean and standard error of the mean (SEM). The mean and SEM presented in text for the H2kb myoblasts-related data is a result of averaging the means and SEMs obtained from the technical replicates of each experiment.

## Results

### The X chromosome locus is associated with fiber number in fast-twitch muscle

**Fig. 1.**
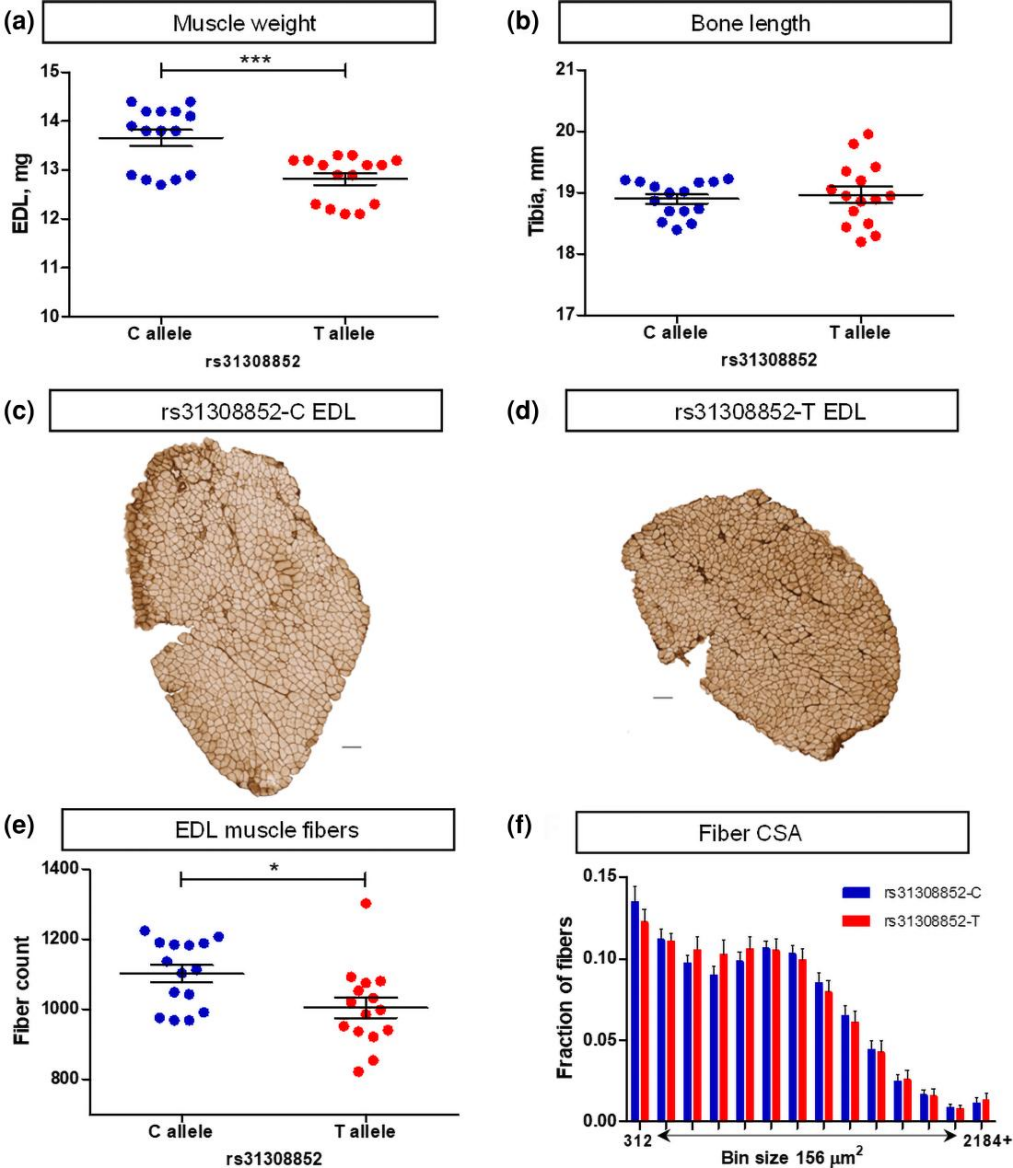
The genotype of the X chromosome locus rs31308852 is associated with the number of EDL muscle fibers in CFW male mice. a) EDL weight. b) Tibia length. c and d) Laminin-stained transverse sections of EDL muscle from the rs31308852-C and rs31308852-T mouse, respectively. Horizontal scale bars 100 µm. e) Fiber number in the EDL muscle. f) Distribution of the cross-sectional area (CSA) of EDL fibers across 156 µm^2^ bins. Data are presented as mean and SEM (*n* = 15 per genotype).

To examine the cellular mechanisms of the effect of the X chromosome locus on muscle weight, we studied the histological properties of the fast-twitch EDL muscle in the CFW carriers of the divergent alleles at a peak marker of the locus, rs31308852. The muscles of the rs31308852-C mice were 7% heavier (*P* = 0.0003) compared with the rs31308852-T allele mice (Fig. 1a). The length of the tibia was similar between the 2 genotypes, 18.9 ± 0.1 vs 19.0 ± 0.1 mm, respectively (*P* = 0.6431) (Fig. 1b). These data demonstrate that the difference in weight was due to the girth of muscle, not the length. The girth can be affected by the number of muscle fibers, their cross-sectional area, or both. The analysis of the histological samples (Fig. 1c and d) revealed that this difference in muscle weight was mediated by 10% more fibers in rs31308852-C mouse muscles compared with rs31308852-T (1,102 ± 25 vs 1,005 ± 30, *P* = 0.0176) (Fig. 1e). The size of the fibers, as assessed by the distribution of fiber cross-sectional area, was similar (Fig. 1f). Thus, the difference in the fast-twitch muscle weight between the genotypes at this X chromosome locus was due to the difference in fiber number.

### *Rps6ka6* affects differentiation of myogenic cells

**Fig. 2.**
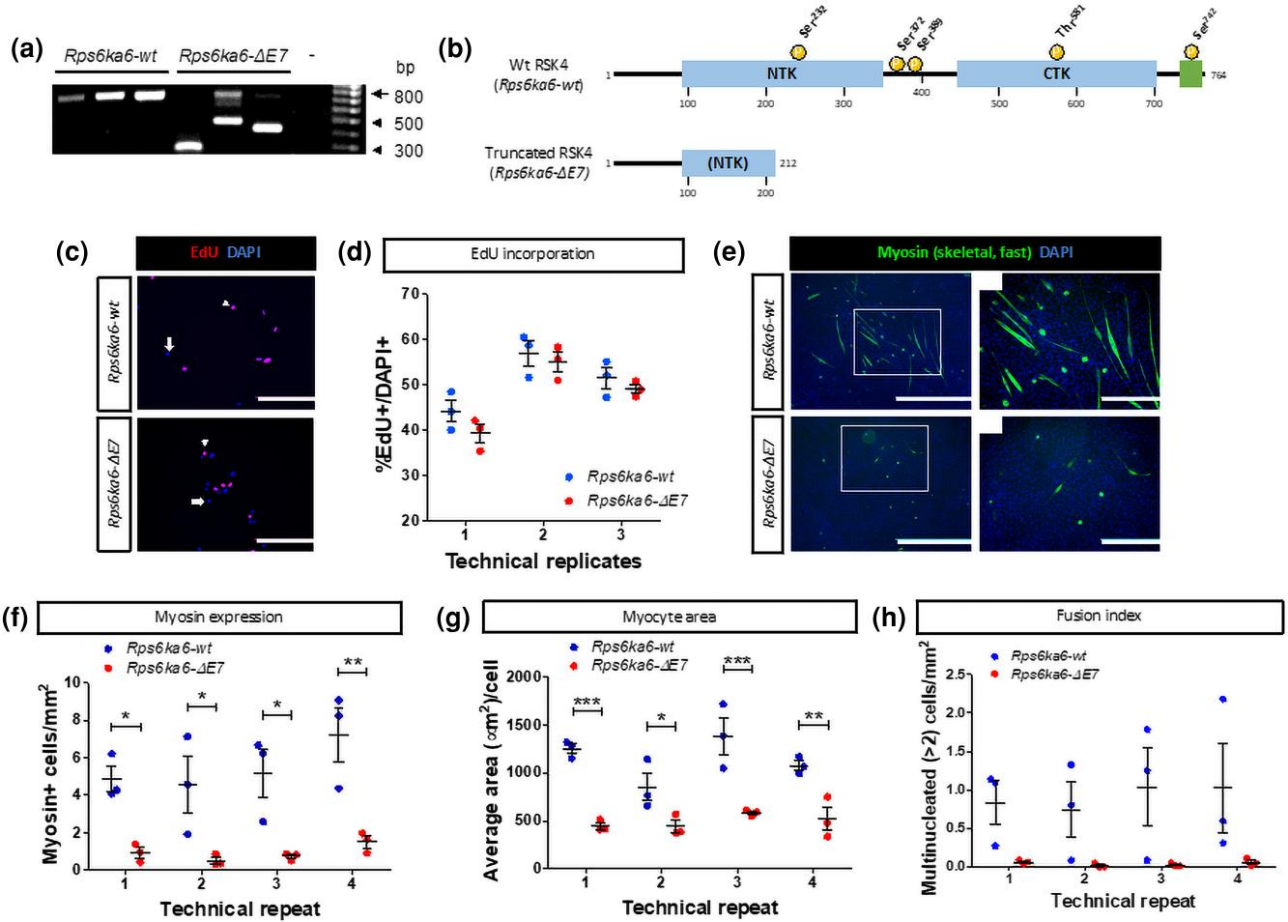
Rps6ka6 is essential for myoblast differentiation but is not required for myoblast proliferation. a) PCR genotyping results of the 3 *Rps6ka6-wt* and 3 *Rps6ka6-ΔE7* clones used in our study (- denotes negative control). b) Schematic representation of the truncated RSK4 protein resulting from the *Rps6ka6-ΔE7* gene. NTK, N-terminal kinase domain; CTK, C-terminal kinase domain; green region, ERK-binding domain. c) Representative images of EdU-treated *Rps6ka6-wt* and *Rps6ka6-ΔE7* myoblasts. Scale bars: 200 μm. d) Quantification of %EdU-positive, proliferating *Rps6ka6-wt* and *Rps6ka6-ΔE7* myoblasts. e) Representative images of myosin-stained myotubes following differentiation of *Rps6ka6-wt* and *Rps6ka6-ΔE7* myoblasts. Scale bars: 1,000 μm in large fields of view and 400 μm in insets. f) Quantification of myosin-positive *Rps6ka6-wt* and *Rps6ka6-ΔE7* cells. g) Quantification of the average area of myosin-positive *Rps6ka6-wt* and *Rps6ka6-ΔE7* cells. h) Quantification of the multinucleated *Rps6ka6-wt* and *Rps6ka6-ΔE7* myosin-positive cells. Data are presented as mean ± SEM (*n* = 3 per genotype).

The rs31308852 is a missense SNP resulting in the Arg45His substitution in the *Rps6ka6* encoded protein. To study the effects of *Rps6ka6* on myogenic lineage, we targeted exon 7 of the gene for excision in H2Kb cells. Three clones of cells with deletion, *Rps6ka6-ΔE7*, and 3 WT clones, *Rps6ka6-wt*, were generated for further analyses (Fig. 2a). Excision of exon 7 creates a premature stop codon (Supplementary Fig. 1), resulting in a severely truncated protein (Fig. 2b). We first quantified the EdU-positive cells to compare the rate of proliferation between the 2 genotypes (Fig. 2c). We were not able to identify a statistically significant difference between *Rps6ka6-wt* and *Rps6ka6-ΔE7* cells, with cumulative average across all 3 repeats of the EdU-positive cells over the total number of cells of 50.8 ± 2.2% vs 47.8 ± 2.5%, respectively (2-way ANOVA, genotype factor, *P* = 0.11) (Fig. 2d). The effect of time (between the 3 consecutive passages of the cells) was statistically significant (*P <* 0.0001) because of the lower proportion of the EdU-positive cells of both genotypes at the first time point (Fig. 2d), but the interaction term was not statistically significant (*P* = 0.7748). The results of this assay reveal no *Rps6ka6* effect on proliferation of the myogenic cells.

Additionally, we assessed the migrative capacity of *Rps6ka6-wt* and *Rps6ka6-ΔE7* myoblasts using an in vitro scratch assay (Supplementary Fig. S2a). We were not able to identify any statistically significant difference between the motility of the *Rps6ka6-wt* (cumulative average of 41.6 ± 4.4% wound closure) and *Rps6ka6-ΔE7* cells (cumulative average of 42.9 ± 2.7% wound closure) (2-way ANOVA, genotype factor, *P* = 0.74) (Supplementary Fig. S2b). Nevertheless, there was some technical variation between consecutive passages (2-way ANOVA, technical repeat factor, *P* = 0.0099) (Supplementary Fig. S2b). These data suggest that variation in the *Rps6ka6* gene does not affect the migrative ability of myoblasts *in vitro*.

We then compared myogenic differentiation between the 2 genotypes (Fig. 2e). Counting the myosin-expressing cells across the 4 time points indicated that they were ∼5-fold more numerous in the *Rps6ka6-wt* cultures compared with the *Rps6ka6-ΔE7* cell cultures (5.4 ± 1.2 vs 0.91 ± 0.22 cell/mm^2^; 2-way ANOVA genotype factor, *P* < 0.0001) (Fig. 2f). The effects of time or the time-by-genotype interaction were not statistically significant (*P* = 0.2384 and *P* = 0.7517, respectively). Myosin-expressing cells were ∼2-fold larger in *Rps6ka6-wt* cell cultures compared with *Rps6ka6-ΔE7* (1,141.5 ± 110.2 vs 499.5 ± 59.4 µm^2^; 2-way ANOVA genotype factor, *P* < 0.0001) (Fig. 2g). Although the myosin-expressing area varied among the consecutive passages (effects of time *P* = 0.0364), the time-by-genotype interaction was not statistically significant (*P* = 0.1943). We then examined the fusion index characterized by the number of cells with more than 2 myonuclei per unit of area. The number of the multinucleated cells in the *Rps6ka6-wt* cell cultures, albeit variable among the clones, was larger compared with the *Rps6ka6-ΔE7* cell cultures (0.91 ± 0.43 vs 0.040 ± 0.018 cell/mm^2^; 2-way ANOVA genotype factor, *P* = 0.0013) (Fig. 2h). The effects of time or the time-by-genotype interaction were not statistically significant (*P* = 0.9464 and *P* = 0.9563, respectively). These data demonstrate that disruption of *Rps6ka6* significantly impairs the differentiation potential of the myogenic cells.

### *Pou3f4* only affects fiber number in slow-twitch muscles

**Fig. 3.**
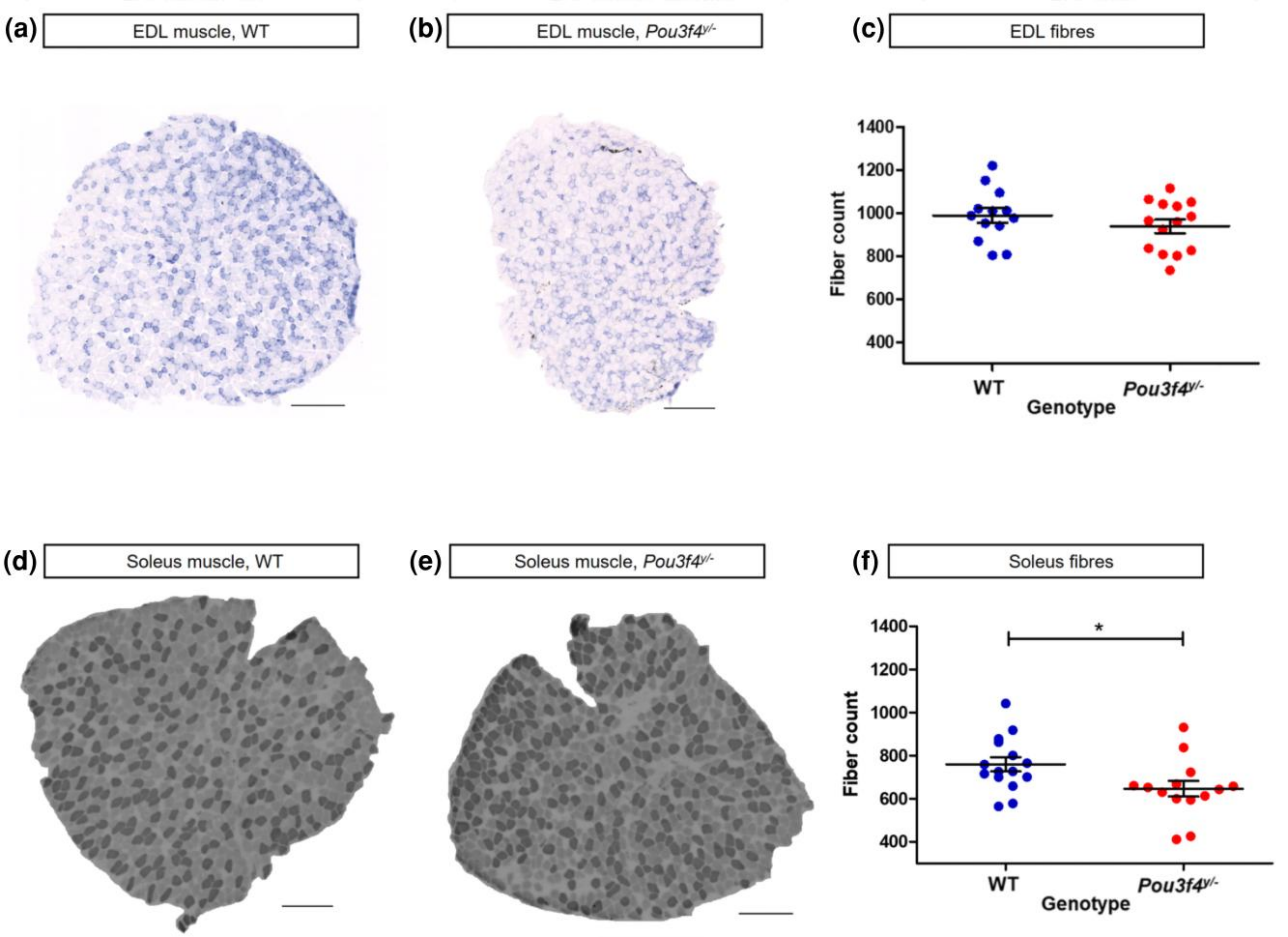
*Pou3f4* ablation leads to a reduction in fiber number in slow- but not fast-twitch muscles. a and b) Micrographs of tetrazolium reductase–stained fast-twitch EDL muscle of WT and *Pou3f4^y/−^* mice, respectively. c) Fiber number in EDL muscle samples. d and e) Micrographs of ATPase-stained slow-twitch soleus muscle of WT and *Pou3f4^y/−^* mice, respectively. f) Fiber number in soleus muscle samples. Horizontal scale bars 200 µm. Data are presented as mean ± SEM (WT, *n* = 15; *Pou3f4^y/−^*, *n* = 14).

To study if *Pou3f4* could be associated with the effect of the X chromosome locus, we analyzed muscle samples of the *Pou3f4*^y/−^ mice and their WT littermates. We focused on the number of muscle fibers because this trait was primarily affected by the rs32308852 genotype in CFW mice and because it is not influenced by the wide age range of mice used in this analysis (Ontell *et al*. 1988). Fiber number in the EDL muscle was similar between the *Pou3f4*^y/−^ and WT mice (938 ± 32 and 987 ± 34, respectively; *P* = 0.2947) (Fig. 3a). The analysis of the slow-twitch soleus muscle showed a 15% reduction in fiber number in the *Pou3f4*^y/−^ muscles compared with the WT (646 ± 36 vs 760 ± 33, *P* = 0.0268) (Fig. 3b). Hence, *Pou3f4* deletion reduces the number of fibers in the slow-twitch soleus muscle but does not affect muscle fiber number in the fast-twitch EDL.

## Discussion

In this study, we examined the cellular mechanisms of the effect of the chromosome X locus associated with variability in fast-twitch muscle mass of CFW mice (Nicod *et al*. 2016) and studied the role of 2 positional candidates, *Rps6ka6* and *Pou3f4*. We found that the locus was primarily associated with ∼10% difference in the number of muscle fibers constituting fast-twitch EDL muscle. The *Rps6ka6* gene played an enabling role in the differentiation of myogenic cells and emerged as the primary candidate for explaining the effect of the locus on the fast-twitch muscle mass. The *Pou3f4* gene did not affect the number of muscle fibers in the fast-twitch muscle but did so in the slow-twitch muscle. Both positional candidates impacted the properties of myogenic tissue.

### Genetic variability affects the number of muscle fibers

In a seminal study, Lexell and colleagues (Lexell *et al*. 1988) documented a 2-fold individual difference in the number of muscle fibers in vastus lateralis of young males. This observation is of clinical importance because the number of fibers declines with aging (Lexell *et al*. 1988) and fewer fibers at a young age may signal an increased risk of sarcopenia, the aging-related decline in muscle mass and function, which erodes the quality of life of the elderly and increases the risk of mortality (De Buyser *et al*. 2016; Beaudart *et al*. 2017). However, that study did not investigate if the difference in the number of fibers is a congenital outcome of, for example, availability of nutrients during prenatal development as demonstrated in rodents (Dwyer and Stickland 1994) or due to inheritance of variants of genes that are relevant in the development, growth, and maintenance of muscle tissue. The findings of our study that the number of fibers is affected by the genotype at the rs31308852 marker suggest that individual differences observed in humans may be an outcome of inherited variants of relevant genes.

### *Rps6ka6* plays a role in myogenic differentiation

The *Rps6ka6* gene encodes the p90 ribosomal S6 kinase 4 (RSK4) protein, 1 of the 4 members of this gene family (Lara *et al*. 2013). The RSK proteins are downstream targets of the extracellular signal regulated kinase (Ras/ERK) pathway. The RSK1–3 proteins have been well characterized, but the function of RSK4 remains poorly understood (Romeo *et al*. 2012). Unlike the 3 other isoforms, RSK4 exhibits a growth factor independent constitutive activity (Dummler *et al*. 2005). The gene has been studied in the context of cancer (Xu *et al*. 2021), but this is the first report on its role in myogenesis of skeletal muscle. A low level of *Rps6ka6* transcript is found in skeletal muscle in humans (Supplementary Fig. S3). This is consistent with the low levels of RSK4 protein in mouse skeletal muscle (Dummler *et al*. 2005). It is primarily a cytosolic protein with a small fraction showing nuclear localization (Dummler *et al*. 2005) and low level of expression in most tissues (Romeo *et al*. 2012). The gene, however, is expressed at a substantial level during embryonic development, and in the organogenesis stage, its expression exceeds the combined level of the RSK1-3 transcripts (Romeo *et al*. 2012). Deletion of *Rps6ka6* severely affects the morphology of the embryo, highlighting its importance during development (Cox *et al*. 2010). A SIFT score for missense SNP rs31308852 in CFW population is predicted to be tolerated for protein function (Ng and Henikoff 2003); however, additional studies are required to establish if this substitution could explain subtle phenotypic effects.

The C2C12 myogenic cell line has been derived from satellite cells and is used as an in vitro model of myogenesis (Yaffe and Saxel 1977; Blau *et al*. 1983). Expression of *Rps6ka6* in C2C12 cells at a myoblast stage is low but increases during differentiation into myotubes, the precursors of muscle fibers (Chen *et al*. 2006). This temporal pattern of expression parallels the findings of our study where mutation of *Rps6ka6* did not affect proliferation of H2Kb myoblasts but did affect their differentiation. Hence, despite the low level of *Rps6ka6* expression in adult tissues, *Rps6ka6* may play a role during embryogenesis and fetal development, at the time when muscle fibers are formed in mice (Ontell *et al*. 1988). The molecular mechanisms of the effect of RSK4 remain unclear.

### Muscle-type specific effects of *Pou3f4*

The *Pou3f4* gene encodes a transcription factor (Mathis *et al*. 1992) implicated in X-linked deafness (DNFX2) in humans (de Kok *et al*. 1995). It was targeted in the present study because in a GWAS on muscle weight in CFW mice, *Pou3f4* was the only annotated gene in the 95% confidence interval of the locus for the TA and EDL muscles (Nicod *et al*. 2016). Furthermore, a previous report implicated this gene in *in vivo* myogenesis (Dominov and Miller 1996). In the present study, however, deletion of the gene did not significantly affect the number of fibers in the fast-twitch EDL muscle but did decrease the number of fibers, albeit only by 15%, in the slow-twitch soleus muscle. It is peculiar though that no association of soleus weight with the X chromosome linked locus was detected in the CFW mice GWAS (Nicod *et al*. 2016). This could be due to a combination of the modest effect of the gene emerging from the *Pou3f4^y/−^* model analysis and the size of the soleus muscle which is ∼40% smaller than EDL in CFW males (Nicod *et al*. 2016). The latter would limit the accuracy of measurements and the statistical power for detection of associations in a GWAS.

The *Pou3f4* gene is expressed in mouse muscle tissue during embryonic days 15–18 (Dominov and Miller 1996) when muscle fibers are actively forming (Chal and Pourquié 2017). The rodent muscle fiber type identity between the slow-twitch and fast-twitch types is acquired at around the same time of fetal development (Schiaffino and Reggiani 2011). The temporal overlap of this developmental process with the expression of *Pou3f4* might be linked to the slow-twitch muscle-specific effect in the *Pou3f4^y/−^* model; however, specific mechanisms remain to be explored. Expression of *Pou3f4* declines postnatally and becomes undetectable in adult muscle, suggesting its primary role in the developmental stage (Dominov and Miller 1996). This temporal pattern of expression may explain why *POU3F4* mutations in DFNX2 patients do not appear to cause noticeable muscle abnormalities (Dominov and Miller 1996).

## Conclusion

We found that the X chromosome locus implicated in the variation of the fast-twitch muscle mass in CFW mice affects the number of muscle fibers. The 2 positional candidate genes, *Rps6ka6 and Pou3f4*, impact the properties of myogenic tissue. *Rps6ka6* affects the differentiation of myogenic cells and might be responsible for the effect of the locus on the weight of fast-twitch muscle. *Pou3f4* affects fiber number in slow-twitch muscle. Hence, both positional candidates of the locus influence skeletal muscle mass through impacting the number of muscle fibers.

## Supplementary Material

jkae046_Supplementary_Data

## Data Availability

The H2Kb cell clones are available upon request. The authors affirm that all data necessary for confirming the conclusions of the article are present within the article, figures, and tables. Supplemental material available at G3 online.
