# Supplementary material for: Validation of positional candidates Rps6ka6 and Pou3f4 for a locus associated with skeletal muscle mass variability
Source: G3 (Bethesda). 2024 Apr 5;14(5):jkae046. doi: 10.1093/g3journal/jkae046 (PMC11075558; doi:10.1093/g3journal/jkae046)
Supplement: jkae046_Supplementary_Data [file jkae046_supplementary_data.docx]

**Supplementary figures**

**
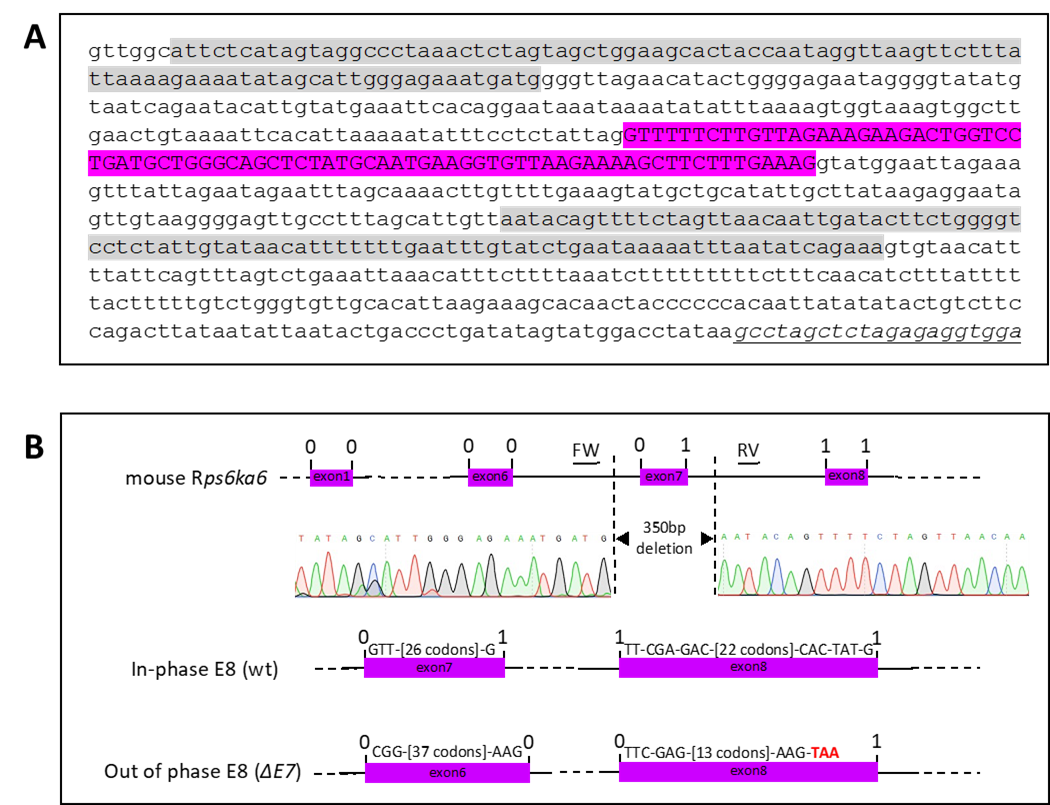
**

**Figure S1. Establishment of the *Rps6ka6-ΔE7* clones.** **A**, the 5’-to-3’ sequence of the *Rps6ka6* exon 7 (ENSMUSE00000152471; in purple capitals) flanked by the introns (lower case). The reverse sequencing primer is italicised and underlined at the 3’ end of the sequence. The Sanger sequencing of the 3G8 clone illustrated here showed 100% identity for 191 intronic bases highlighted in grey but was interrupted by a 4-base long mismatch (not shown) replacing ~350 bp of the native sequence. **B**, at the top purple boxes represent exons and black lines represent introns. The phases of each exon are denoted by the numbers at the beginning and end of the exon box. The primers used for the PCR amplification (forward, FW, and reverse, RV) are indicated alongside the length of the introns flanking exon 7. In wild-type cells (middle) exon 7 ends in phase 1 allowing for an in-frame reading of exon 8, also starting in phase 1. In contrast, in *Rps6ka6-ΔE7* cells (bottom) exon 6 ends in phase 0 leading to a frameshift mutation of exon 8 which now starts in phase 0 instead of 1. Consequently, a nonsense mutation in exon 8 results in a truncated RSK4 protein being expressed. Individual codons are denoted on top of the exon boxes and are separated by dashes. The premature stop codon is highlighted in red.


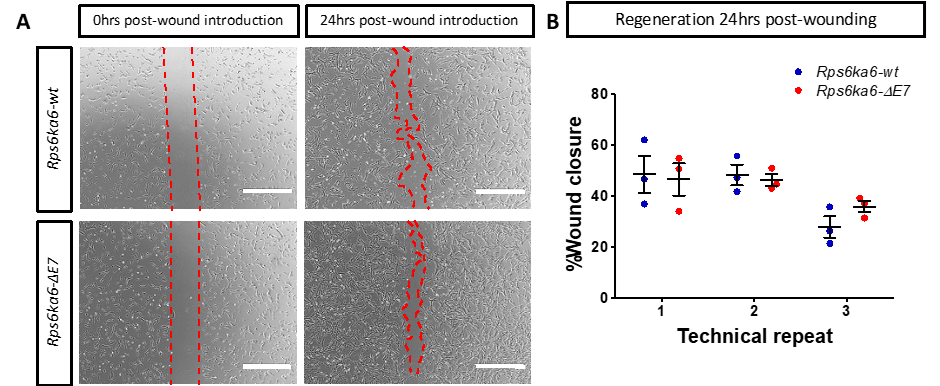


**Figure S2. Migrative capacity of *Rps6ka6-wt* and *Rps6ka6-ΔΕ7* myoblasts. A,** representative images of *Rps6ka6-wt* (top) and *Rps6ka6-ΔΕ7* (bottom) myoblasts immediately after (left) and 24 hours post-wounding (right). Scale bars: 100μm. **B**, quantification of the percentage wound closure. Data presented as mean ± SEM.


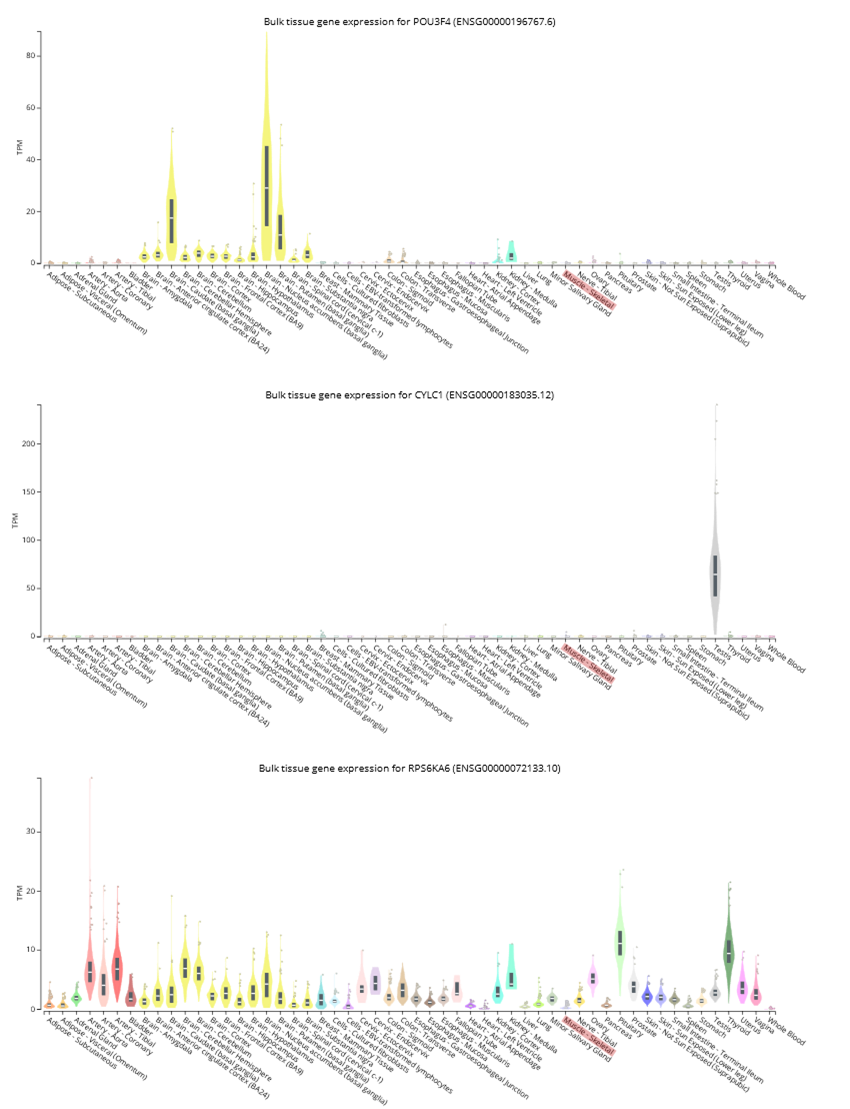


**Figure S3. Expression of *POU3F4*, *CYLC1* and *RPS6KA6* human tissues.** Skeletal muscle is highlighted in red. Gene expression is presented as Transcripts Per Million (TPM). Median expression values for *POU3F4*, *CYLC1* and *RPS6KA6* genes across 803 skeletal muscle sample were 0.0000, 0.0000 and 0.1821 TPM, respectively. The plots in this figure were obtained from the GTEx Portal on 06/01/23 (dbGaP Accession phs000424.v8.p2).
